# ARIA–Italy managing allergic rhinitis and asthma in a changing world: The role of the Pharmacist^☆^

**DOI:** 10.1016/j.waojou.2025.101055

**Published:** 2025-04-25

**Authors:** Giovanni Paoletti, Corrado Giua, Alessandro Marti, Matteo Alberto Baio, Nicolò Valli, Erminia Ridolo, Maria Teresa Ventura, Giovanni Passalacqua, Francesca Puggioni, Olga Lourenço, Jean Bousquet, Giorgio Walter Canonica, Enrico Heffler, Carlo Lombardi

**Affiliations:** a Department of Biomedical Sciences, Humanitas University, Pieve Emanuele, Milan, Italy; b Personalized Medicine, Asthma and Allergy, IRCCS Humanitas Research Hospital, Rozzano, Milan, Italy; c Società Italiana Farmacia Clinica (SIFAC), Viale Regina Margherita 30, 09124 Cagliari, Italy; d Department of Medicine and Surgery, University of Parma, Italy; e University of Bari Medical School, Bari, Italy; f Institute of Sciences of Food Production, National Research Council (ISPA-CNR), Bari, Italy; g Allergy and Respiratory Diseases, Department of Internal Medicine (DIMI), University of Genoa, 16132 Genoa, Italy; h IRCCS Policlinico San Martino, 16132 Genoa, Italy; i Faculty of Health Sciences and CICS - UBI Health Sciences Research Centre University of Beira Interior Covilhã Portugal, Portugal; j Institute of Allergology, Charité-Universitäts medizin Berlin, Corporate Member of Freie Universität Berlin and Humboldt-Universität zu Berlin, Berlin, Germany; k Departmental Unit of Allergology, Immunology & Pulmonary Diseases, Fondazione Poliambulanza, Brescia, Italy; l Fraunhofer Institute for Translational Medicine and Pharmacology ITMP, Immunology and Allergology, Berlin, Germany; m ARIA, Montpellier, France

**Keywords:** Pharmacists, Allergic, Rhinitis, Asthma, Patient care management, Patient care team

## Abstract

Allergic rhinitis (AR) and asthma are common respiratory disorders that often occur together, affecting quality of life and increasing healthcare expenses of patients.

These chronic illnesses are often managed without medical supervision, creating distinct challenges. A lack of resources can limit regular follow-up, which in turn promotes disease mismanagement and an increased reliance on self-medication, including the inappropriate use of corticosteroids and nasal decongestants. Community pharmacies could serve as critical primary healthcare providers, facilitating AR and asthma management by promoting therapy adherence, minimizing drug misuse, and improving symptom monitoring using digital tools.

The evolving role of pharmacists as vital healthcare team members is highlighted by their involvement in screening, prevention, and patient education, particularly in underserved communities. Strengthening the partnerships between pharmacists, physicians, and patients may lead to more tailored and effective management strategies. This collaborative approach has demonstrated promise in enhancing disease outcomes and reducing healthcare costs.

## Introduction

Allergic rhinitis (AR) and asthma are chronic respiratory conditions that often occur together and significantly impact global health.^1^, ^2^, ^3^ Both situations involve an immune-mediated inflammatory response in the airways. AR is an inflammation of the nasal mucosa caused by exposure to allergens, leading to symptoms like sneezing, runny nose, nasal congestion, and itching.^4^^,^^5^ On the other hand, asthma affects the lower respiratory tract, causing episodes of wheezing, shortness of breath, chest tightness, and coughing, often triggered by allergens, respiratory infections, or environmental factors.^6^

AR affects 10–40% of the global population,^7^, ^8^, ^9^ and its prevalence is increasing, especially among children and adolescents.^10^ In Italy, the prevalence of AR ranges between 5% and 35%,^11^, ^12^, ^13^, ^14^ with significant regional variations due to environmental factors such as pollution and allergen exposure.^15^, ^16^, ^17^ Recent studies show a rising trend in physician-diagnosed AR, with rates nearly doubling in the last decade.^9^ Asthma also has high prevalence rates, particularly in urbanized and industrialized regions with significant pollutant exposure. Epidemiological data suggest that 40% of patients with persistent rhinitis also have asthma, highlighting the interconnected nature of these conditions.^10^^,^^18^

The global burden of AR and asthma is substantial, with socioeconomic implications. While not always classified as a severe disease, AR can significantly affect quality of life,^19^ resulting in reduced productivity, school absenteeism,^20^ and impairments in work performance.^21^ The economic cost of AR is substantial due to both direct healthcare expenditures and indirect costs related to lost workdays and decreased productivity. The Total Cost of Allergic Rhinitis (TOTALL) study emphasized this disease's previously underestimated economic burden, showing that indirect costs, including presenteeism (reduced productivity while at work), play a crucial role.^22^

Both AR and asthma significantly reduce the quality of life (QoL) of patient and affect their ability to perform daily activities. AR can cause sleep disturbances, fatigue, and cognitive impairments, worsening QoL. Studies have shown that individuals with AR have an increased risk of developing anxiety and depression,^23^, ^24^, ^25^ adding to the social and psychological burden of the disease. Additionally, AR often coexists with other conditions, such as chronic rhinosinusitis, otitis media, and conjunctivitis, making its management more complex.^26^

Asthma poses a more immediate health threat, particularly in severe cases that can lead to hospitalization and even death if not properly controlled. The economic and social impact of asthma is also significant, with high emergency department visits and hospital admissions rates, especially during peak allergen seasons or pollution events.^27^ Both conditions are exacerbated by environmental factors such as pollution and climate change,^28^ with evidence linking air quality deterioration to increased incidence and severity of AR and asthma.

The increasing prevalence of AR and asthma, particularly among urban populations, highlights the need to enhance public health strategies that prioritize prevention, early diagnosis, and comprehensive management.^29^ Addressing the socio-economic and quality-of-life impacts of these diseases requires coordinated efforts to decrease exposure to allergens and pollutants, enhance patient education, and encourage adherence to treatment guidelines.

Most economies need help to deliver modern health care effectively. There is a need to support the transformation of the health care system into integrated care with organizational health literacy. Integrated care pathways are structured multidisciplinary care plans which detail essential steps in the care of patients. They promote the translation of guidelines into local protocols and their subsequent application to clinical practice. They empower patients and their carers (health and social). Integrated care pathways (ICPs) differ from practice guidelines as a multidisciplinary team utilizes them and have a focus on the quality and coordination of care. Allergic Rhinitis and its Impact on Asthma (ARIA) proposed real-life ICPs centered around the patient with rhinitis and/or asthma, and using mHealth (eg, MASK-air) including environmental exposure.^30^ Pharmacists are at the forefront of ICPs for AR. An ICP is intended to act as a guide to treatment.^31^^,^^32^ The first ARIA guideline in the pharmacy was published in 2004 stressing the role of pharmacists in the management of AR.^33^ It became more important as the number of over-the-counter (OTC) drugs for AR increased. However, there are large differences concerning the role of pharmacists between countries.

## Difficulties of the physicians in managing patients with rhinitis and asthma

In an ideal world, the correct management of asthma and AR should include adequate drug therapy and global patient care, with a holistic multidisciplinary approach considering the various interactions between pathologies and therapies. However, in many countries, the long-term management of chronic conditions such as asthma and AR is not always feasible due to limited financial resources. This often leads to a loss of patient follow-up, resulting in poor disease control. Various worldwide studies have proposed strategies to improve the management of chronic patients, such as increasing the availability of community healthcare services and ensuring that medications are dispensed by qualified personnel, highlighting the importance of close collaboration between doctors and pharmacists.^34^ In Italy, the decentralized structure of the healthcare system results in disparities in the quality of care based on where patients receive health services. This means that patients may only sometimes get the necessary follow-up and continuous therapy adjustments according to their needs. The lack of coordination between hospital and territorial services leads to suboptimal management of chronic patients.^35^

These organizational shortfalls often push patients toward self-medication, increasing the risk of drug misuse. The most commonly abused medications by asthmatic patients are oral corticosteroids, whose chronic use can lead to severe systemic side effects such as osteoporosis, diabetes, and hypertension. Improper management of these drugs can also result in adrenal suppression and the development of cardiovascular diseases.^36^^,^^37^ Similarly, patients with AR often find relief through the improper use of intranasal sympathomimetic decongestants, as also recently evidenced in a pharmcy study from Belgium by Schiere et al,^38^ which not only leads to dependency but also causes the so-called rhinitis medicamentosa and damage to the nasal mucosa.^39^ A study conducted through surveys in Italian pharmacies confirmed that 84·8% of patients prefer self-medication with sympathomimetic decongestants.^40^ On the other hand, the medications most frequently recommended by pharmacists for rhinitis are predominantly isotonic/hypertonic solutions, followed by sympathomimetic amines and topical steroids. These findings underscore the need for continued educational efforts aimed at both pharmacists and patients to promote the rational use of medications for allergic rhinitis, with particular attention to limiting the inappropriate or prolonged use of intranasal sympathomimetic amines, which remain widely utilized despite their known adverse effects. The study also highlighted that the primary purchasers of intranasal sympathomimetic amines are those with symptoms attributable to AR.

These observations are further substantiated by the report on non-prescription drug sales in Italy.^41^ In 2022, approximately 110 million units of respiratory medications were purchased, underscoring the increasing complexity of managing these conditions. Of this expenditure, 3·6% was allocated to anti-allergic and antihistamine drugs. The 52·5% of the costs involved cold medications, including nasal decongestants. despite these medications are indicated for acute conditions, they are often, as mentioned above, misused by patients.

Data from the international survey on the management of allergic rhinitis (ISMAR) registry^42^ and surveys conducted by the European Academy of Allergy and Clinical Immunology (EAACI)^43^ indicate that patients tend to seek medical consultation based on the severity and duration of their symptoms. Both patients with mild and moderate/severe rhinitis often begin by attempting to manage their condition with non-prescription drugs, such as nasal decongestants and oral antihistamines (90·6% and 93·2%, respectively). Patients are prompted to consult a physician when they are unable to control symptoms despite self-medication, or the presence of asthma as a comorbidity, which necessitates a more holistic approach to patient care.

These data are once again mirrored in the sales reports.^41^ In fact, in 2022, among non-prescription antiallergic drugs, only 32% were recommended by a healthcare professional such as a doctor or pharmacist. So, most drugs sold to allergy patients are self-medication. Furthermore, according to market projections, this condition will have a positive trend in the coming years in favor of self-medication.

Another challenge in managing patients with respiratory allergies is helping them understand the importance of consistent adherence to treatment. Results from a study based on surveys of patients with AR^44^ revealed that the average adherence rate to therapy as prescribed was 58·9%, varying according to the severity of perceived symptoms. Data from the Asthma Patients' and Physicians' Perspectives on the Burden and Management of Asthma (APPaRENT) 1 and APPaRENT 2 survey^45^ indicate that adherence to maintenance inhalation therapy with inhaled corticosteroids (ICS) in patients with bronchial asthma ranges between 60·5% and 70·8%. Lower compliance rates were observed in younger patients and those with mild to moderate asthma, leading to a tendency to rely more on emergency medications rather than on maintenance therapy aimed at reducing airway inflammation.

The study also highlighted a tendency among general practitioners to prescribe only short-acting beta-agonists (SABA) and a patient dependence on these drugs. In fact, 66·6% of patients who were prescribed a treatment protocol involving both ICS/long-acting beta agonist (LABA) for both as-needed and maintenance therapy still purchased a short-acting beta agonist (SABA) inhaler without a medical prescription. Another factor contributing to poor asthma control is improper inhaler technique. A study on asthmatic and chronic obstructive pulmonary disease (COPD) patients^46^ showed that incorrect use of inhaler devices was identified in 53% of patients using metered-dose inhalers (MDIs) and in 39% of those using Dry Powder Inhaler (DPI). For this reason, it is always advisable to suspect poor adherence to therapy or incorrect inhaler technique in patients with respiratory allergies when symptoms do not improve and to emphasize to patients the benefits of following the prescribed treatment regimen correctly. In this context, the pharmacist could be a central figure in managing patients suffering from chronic diseases (See Table 1).

**Table 1:** This table summarizes in 4 points the potential unmet needs of asthmatic and rhinitis patients which could be improved with the help of the pharmacist

Potential unmet needs for asthmatic and rhinitis patients that can be improved with the pharmacist's help	
**Patient education**	○ Difficulty in educating patients about the connection between rhinitis and asthma, and how managing one can improve the other. ○ Challenges in providing clear and accessible information on self-management strategies, especially for patients with low health literacy
**Patient adherence**	○ Non-adherence to treatment plans is common, particularly with long-term therapies like inhalers or allergy medications. ○ Ensuring patients understand the importance of consistent treatment even when symptoms subside.
**Complex treatment regimens**	○ Managing 2 chronic conditions often requires multiple medications, which can be overwhelming for patients. ○ Difficulties in ensuring adherence to inhalers, nasal sprays, and oral medications.
**Impact of comorbidities**	○ Patients with rhinitis and asthma often have other related conditions (e.g., eczema, sinusitis), complicating management. ○ The need for holistic treatment plans that address all comorbidities. ○ Possible side effects of treatment on other comorbidities (e.g. topical nasal corticosteroid therapy in glaucoma patients)

## The pharmacy as a first point of access for citizens to health care

In Europe, two-thirds of the population can access a pharmacy in under 5 min, and 98% can do so within 30 min.^47^

Italy has 20,979 pharmacies distributed proportionally across the national territory based on population density, a key criterion for ensuring medication access.^48^^,^^49^ The placement of pharmacies is defined by a specific plan, a regional administrative act that serves as the fundamental tool for determining the distribution of pharmacies across the territory, mainly based on citizens' numbers. This system ensures a widespread and well-distributed network of pharmacy locations that meet citizens' needs. The 2012 reform (Law No. 27/2012, Art. 11) established the opening of 1 pharmacy for every 3300 inhabitants, lowering the previous threshold. It is also delegated to municipalities, in coordination with local health authorities and the relevant pharmacy board, the task of defining areas where pharmacies can be located to ensure equitable distribution. Many municipalities have taken advantage of the reform's provision allowing the opening of new pharmacies in underserved areas or areas with high foot traffic, such as ports, airports, train stations, and shopping centers, regardless of population density.^50^ As a result, Italy has 1 pharmacy for every 2938 inhabitants, slightly above the European average of 1 pharmacy per 3237 inhabitants.^48^ The same reform also allows pharmacy managers to offer services during additional hours and periods beyond the mandatory opening hours, provided prior notice is given to the competent health authority.^50^

The busiest times for pharmacies are early morning and evening, aligning with the daily work routines of patients. Many pharmacies are also open on Saturdays, and often on Sundays and holidays as well.^48^ Mandatory shifts ensure that 1800 pharmacies are open during nighttime hours, with additional pharmacies voluntarily participating in overnight services. It is estimated that 2% of total medication purchases occur during these nighttime hours.^48^

This widespread and convenient pharmacy access aligns with patient preferences for easily reachable healthcare touchpoints, particularly outside of standard working hours. Several studies have shown that patients often consult pharmacists due to their extended availability, perceived approachability, and the possibility of receiving immediate advice without needing an appointment.^51^^,^^52^

The COVID-19 pandemic further highlighted the role of pharmacists as key points of reference for citizens. During the emergency, there was an increase in inquiries to pharmacists about medications, therapies, and health information in general. Pharmacies remained open throughout the lockdown, with appropriate safety measures in place, leading to an increased perception of trust in this professional figure even after the emergency ended.^53^ These factors, combined with the convenience of accessing pharmacies without an appointment, resulted in 4 million daily visits to pharmacies in 2023,^48^ making pharmacies the primary point of access to healthcare for citizens, both in Italy and across Europe.^47^ In the Italian context, where there are 18 million annual emergency room visits, 4 million of which are deemed inappropriate^54^ and a decreasing number of General Practitioners, now totaling around 40,000 — down by approximately 2000 between 2019 and 2021^55^ — it is increasingly important for pharmacists to be involved and trained as part of a multidisciplinary team alongside physicians and other healthcare professionals. This approach will help improve healthcare quality of citizens.^47^^,^^56^

## Evolution of the concept of pharmacy from place of dispensing to service pharmacy

The concept of pharmacies serving as a point of care is now widely accepted, but it was almost unthinkable until recently. In the 1980s and 1990s, community pharmacies primarily focused on medications, with their main tasks including preparing, dispensing, and providing counseling related to pharmaceuticals.^57^ This allowed them to establish themselves as genuine “houses of medicine”.

The clinical healthcare environment has rapidly evolved over the years, requiring a more prepared and efficient territorial healthcare system. These changes have significantly impacted pharmacies, turning them into fully-fledged first-line healthcare hubs that work closely with medical professionals to address health needs promptly.

A significant factor contributing to this shift was undoubtedly the pandemic era. The public health emergency required increasing support from community pharmacies to relieve the strain on hospitals, which were dealing with the most critical situations.^58^^,^^59^

Pharmacies are crucial in addressing healthcare challenges due to their widespread presence, open access, and trusted relationships with the public. They can provide essential healthcare services like screening, testing, and vaccinations,^60^, ^61^, ^62^ reaching even those members of the population who are hard to reach or less likely to seek care in other healthcare facilities.

Pharmacy curricula in Italian universities have been progressively updated to reflect this shift, emphasising clinical competencies such as medical history-taking, patient counseling, and the ethical and legal principles underpinning confidentiality and therapeutic decision-making. These competencies are integrated through theoretical modules and practical training, preparing pharmacists to support patients with chronic conditions like AR, chronic rhinosinusitis (CRS), and asthma.

In addition to foundational education, Italian pharmacists are required to engage in continuing professional development (CPD) throughout their careers. National and regional CPD programs now include targeted content on respiratory diseases, inhaler techniques, pharmacovigilance, and interprofessional collaboration. Moreover, international platforms such as the European Forum for Research and Education in Allergy and Airway Diseases (EUFOREA) e-academy provide accessible, evidence-based online learning opportunities, including an introductory level specifically designed for pharmacists.^63^ These modules offer updated knowledge on AR, CRS, and asthma, aligning with ARIA and Early Pregnancy Observation Study (EPOS) guidelines, and represent a valuable resource for newly qualified and practicing pharmacists seeking to expand their clinical role within multidisciplinary care models.

Once primarily seen as a chemist and molecule expert, the pharmacist has now taken on a new role as a clinician with responsibilities that extend beyond merely dispensing.^64^ Their profession now involves providing a genuine “service” focused on patient care.^65^^,^^66^

Pharmacists collect medical histories from patients to guide their healthcare journeys. They promote preventive care for healthy individuals^67^ and manage chronic conditions through initial assessments and telemedicine.^68^, ^69^, ^70^, ^71^ They complement clinical data with validated questionnaires to identify potential care plan issues, enabling personalized educational interventions and fostering synergy among healthcare professionals.^72^

Pharmacists also conduct medication reviews to identify and prevent adverse reactions and drug interactions, contributing to active pharmacovigilance and ensuring appropriate therapy monitoring.^73^, ^74^, ^75^ The main objective is to simplify care routines. It is crucial to regularly assess the safety of medications and potential interactions, especially for patients on multiple medications, in order to encourage deprescribing and make treatment regimens simpler. This can ultimately improve adherence to therapy.^76^

The role of pharmacists as researchers is becoming more prominent. They participate in observational studies prospective,^59^^,^^77^, ^78^, ^79^, ^80^ collect real-world data, and generate evidence valuable to the scientific community. This helps integrate clinical practice in a multidisciplinary framework and allows collaboration with other healthcare and academic excellence centers.

The different aspects described are all interconnected parts of the evolving Italian community pharmacy. It is increasingly aligned with a healthcare model that supports individuals on multiple levels throughout their health journeys. However, this opportunity also poses a challenge, as pharmacies have had to undergo significant structural and logistical changes to meet regulatory standards. They also need to acquire specific skills through certified training to keep pace with the professionalization of the field, following the example of other successful European models.

## The pharmacist who brings multidisciplinary approach to managing rhinitis and asthma

Recent literature suggests that effectively managing chronic conditions like allergic rhinitis and asthma requires a multidisciplinary approach involving collaboration among healthcare professionals.^81^, ^82^, ^83^, ^84^ For instance, in cases of allergic rhinitis, patients often delay seeking medical advice and may start self-treatment instead.^85^

Pharmacists are often the first healthcare providers that patients consult, especially in the early stages of symptoms.^86^ It is not uncommon for patients to feel more comfortable confiding in them rather than a physician.^83^^,^^87^ This tendency is partly due to the wide availability of OTC medications, such as antihistamines and nasal sprays, which can be purchased without a prescription.^88^

In this context, the pharmacist has to verify the appropriateness of the dispensed medications, suggest treatments following the most recent guidelines, and investigate any potential side effects.^89^ Additionally, they must discourage inappropriate therapies, often self-initiated by patients, such as the overuse of oral corticosteroids or nasal decongestants.^40^^,^^90^

It is important to use therapeutic devices, such as inhalers and nasal sprays, correctly to maximize the effectiveness of treatment. Pharmacists can offer valuable support by educating patients on proper techniques, even using placebo samples, to reduce errors affecting treatment efficacy.^91^

Patients with chronic conditions have to adhere to their therapy consistently. Often, patients tend to underestimate their symptoms, leading to non-adherence to prescribed medication and only taking medication when their symptoms worsen. Pharmacists need to stress the importance of sticking to their therapy. They can also recommend keeping a daily symptom diary to monitor their symptoms. Studies have shown that mobile phone apps can effectively monitor patients' clinical condition and raise disease awareness.^3^^,^^92^^,^^93^

The pharmacy plays a crucial role in managing chronic respiratory disorders by identifying untreated or poorly managed conditions early, monitoring ongoing therapies, and recognizing situations that require medical evaluation. The pharmacist's role should not be viewed as simply an intermediary between the patient and physician, but as an active stakeholder in the multidisciplinary network, contributing to managing these conditions. Due to their closer relationship with the patient, pharmacists improve treatment adherence and enable a more personalized approach to managing these conditions.^84^^,^^89^^,^^94^^,^^95^

## Pharmacist's management flow chart

**Fig. 1:**
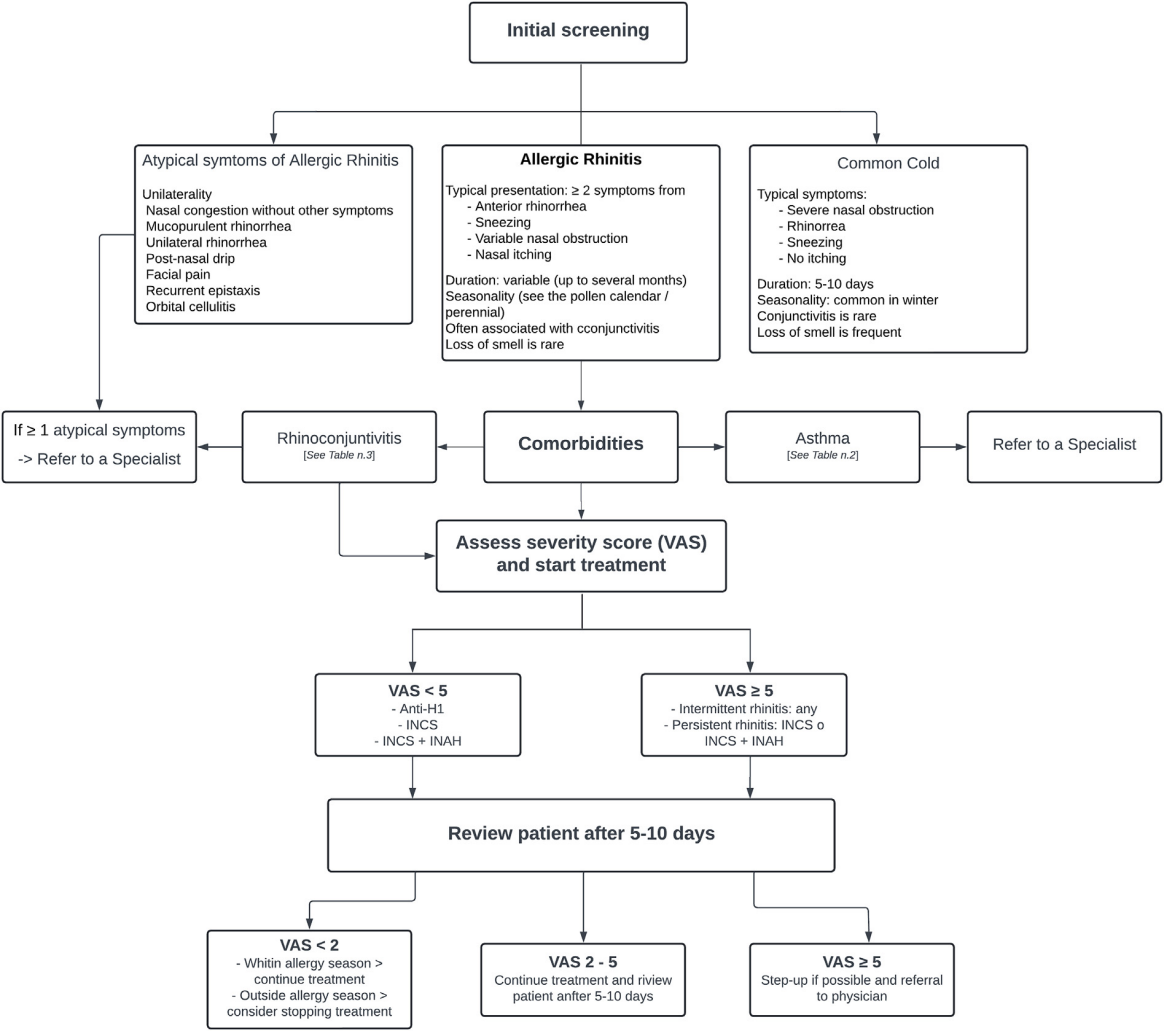
Treatment of Allergic Rhinitis in the pharmacy. Anti-H1, Antihistamine H1 receptor antagonist; INCS, Intranasal Corticosteroid; INAH, Intranasal Antihistamine (Adapted from^89^).

These steps could follow a valid protocol for managing patients with AR, carried out by the pharmacist (see Fig. 1).

### Initial assessment

The pharmacist gathers information about the patient's symptoms during the initial consultation and conducts an initial screening. A comprehensive interview enables the pharmacist to differentiate between a condition that can be managed with over-the-counter (OTC) medications and requires specialized medical intervention.^84^^,^^96^ It is essential to determine whether the patient's symptoms align with allergic rhinitis or may be linked to other conditions, ranging from the common cold to more severe illnesses.^89^ The pharmacist has to be able to identify unusual characteristics, such as one-sided symptoms, isolated nasal congestion, mucus discharge, or the presence of pain or persistent nosebleeds, which should prompt a specialist assessment.^96^

### Comorbidities

When treating allergic rhinitis, the pharmacist has investigated the presence of frequently associated comorbidities. Among these, conjunctivitis and asthma are particularly important to consider:^97^

- Asthma [see Table 2]: When a patient with allergic rhinitis experiences asthma symptoms, they should seek medical evaluation. Allergic rhinitis is known to be a risk factor for the development of asthma, and studies have shown that the 2 conditions often coexist. If the patient has already been diagnosed with asthma, it is important to ensure that both conditions are well controlled. Recent studies have shown that poor control of 1 condition can negatively affect the other. If a patient shows symptoms suggesting a new asthma diagnosis or poor asthma control, it is advisable to recommend a specialist evaluation.^4^^,^^89^^,^^98^

**Table 2:** Screening of rhinitis patients at the pharmacy (Adapted from^96^).

Patient without a diagnosis of asthma	Patient with a diagnosis of asthma
• Have you ever had asthma? • Have you had any asthma symptoms in the past 12 months? • Are you currently taking any medications, including inhalers, aerosols, or tablets for asthma? • Have you experienced wheezing or a whistling sound in your chest at any time in the past 12 months? • Have you had wheezing or whistling when you didn't have a cold? • Have you been short of breath when the wheezing sound was present?	• Have you had difficulty sleeping due to asthma symptoms? • Have you experienced your usual asthma symptoms during the day? • Has your asthma interfered with your usual activities (e.g., household chores, work, or school)? • Do you need your relief inhaler more than once a day?
***≥ 1 positive response → probable asthma***	***≥ 1 positive response → poorly controlled asthma***
- Conjunctivitis [see Table 3] shares the allergic causes of rhinitis and often follows a similar course. It typically responds to symptomatic treatment, but it's important to refer the patient to specialists if they experience atypical symptoms (such as unilateral presentation, photophobia, or a burning sensation without itching) or if symptoms persist despite treatment.

**Table 3:** Screening of typical and atypical symptoms of allergic conjunctivitis at the pharmacy (Adapted from^96^).

Typical symptoms of allergic conjunctivitis	Atypical symptoms of allergic conjunctivitis
- Symptoms associated with rhinitis - Bilaterality - Eye itching - Tearing - Eye redness - No photophobia	- Symptoms not associated with rhinitis - Unilateral conjunctivitis - No eye itching, but burning/pain - Dry eyes - Photophobia

### Severity

It is crucial to objectively assess symptom control and their impact on the patient's daily life.^87^^,^^88^ If symptoms disrupt the patient's sleep or cause significant issues that negatively affect daily activities (work, school, sports, leisure), allergic rhinitis is classified as moderate to severe and uncontrolled.^4^ Therapy escalation is recommended in such cases, and a medical re-evaluation may be necessary.

One effective way to objectively assess the intensity of nasal and ocular symptoms perceived by the patient is by using the Visual Analog Scale (VAS). The VAS is simple and quick, making it a useful tool for pharmacists to track a patient's progress over time.^99^ Numerous studies have found it an effective and reliable tool for guiding potential treatment adjustments.^83^^,^^88^^,^^89^

### Treatment and patient education

Pharmacists play a key role in patient education, particularly when multiple medications are involved. One of their primary responsibilities is to provide clear and accurate information about therapeutic benefits, potential side effects, and proper usage of each treatment option. By fostering informed adherence, pharmacists contribute substantially to the success of allergy and asthma management plans.

When dealing with untreated allergic rhinitis, pharmacists can help the patient by following good practices and choosing the right therapy, sticking to the more recent guidelines. A significant intervention is to adopt measures to avoid allergens.

In the pharmacy setting, recommendations for allergen avoidance are generally based on non-specific yet practical strategies that can be applied even without formal allergy testing. These include daily nasal saline irrigation, protective face masks during high pollen periods, frequent indoor environment ventilation, and reduced exposure to visible dust or known irritants. While these general measures may relieve symptoms, they are particularly important in initial management or self-care settings.

Furthermore, the pharmacist should always prompt patients to perform nasal irrigation with a saline solution daily, which could be enough for mild symptoms and a useful habit to fulfil just before other topical medications to increase efficacy. When suggesting a treatment, the pharmacist has a multitude of drugs to choose from. Intranasal corticosteroids have been proven to be the most effective single maintenance therapy for AR. In addition to them, it could use oral antihistamines (preferring non-sedating molecules), intranasal antihistamines, mast cell stabilizers, and leukotriene receptor antagonists.^100^ Every time a new therapy is started, the patients should be reassessed in 5 to 10 days.^96^

Pharmacists should always discourage unsafe therapies (such as long-term nasal decongestant sprays or systemic glucocorticoids) and remind the patient that it is advisable to seek a specialist consultation in more severe cases. If the patient is already undergoing treatment, the pharmacist can evaluate its effectiveness, monitor the patient's adherence, and ensure that medications are being administered correctly.^87^^,^^88^

Patients need to understand that some medications take time to become effective, and that consistently following the prescribed therapy is crucial for successful treatment.^87^^,^^89^ One of the main reasons for treatment failure is poor adherence, especially with nasal corticosteroids. Many patients stop treatment early if they do not see improvement after a few days.^101^ In these cases, the pharmacist can explain the long-term benefits of using the therapy continuously and consistently.^96^

With appropriate training, pharmacists could also provide more individualized advice to patients with a known allergic sensitization. In such cases, reinforcing allergen-specific avoidance strategies—targeting, for example, dust mites, pollens, animal dander, or occupational allergens—could improve disease control and support better long-term outcomes. Pharmacists could thus serve as an accessible reinforcement point for specialist-provided instructions, enhancing patient adherence to avoidance measures in daily life.

Moreover, occupational allergy would represent a key area of pharmacist education. Pharmacists could be trained to recognize warning signs of work-related rhinitis or asthma and to recommend timely referral to an occupational health specialist or allergist. Early identification and appropriate guidance might help prevent the progression from occupational rhinitis to occupational asthma, particularly in high-risk individuals.

In addition, pharmacists should be trained to identify individuals with persistent or moderate-to-severe allergic rhinitis who might benefit from allergen immunotherapy (AIT), particularly in younger patients where early intervention may prevent asthma development. Evidence supports the preventive potential of AIT when initiated early in life. Therefore, pharmacists could play a pivotal role in recognizing suitable candidates and referring them promptly to allergy specialists. Incorporating this referral process into pharmacy-based care protocols would strengthen multidisciplinary collaboration and optimize long-term outcomes for allergic patients.^102^, ^103^, ^104^

Finally, in Italy pharmacists do not routinely perform diagnostic or follow-up measurements such as spirometry or peak expiratory flow. However, they may advise patients on proper use of home monitoring devices and support self-monitoring as part of therapeutic education.

### Monitoring and follow-up

It is important to follow up with patients over time to evaluate their ongoing treatment's effectiveness and reassess them after any changes to their therapy. Reassessment should take place 5–10 days after starting or adjusting treatment.

Several studies have shown that mobile phone apps can effectively monitor clinical conditions of patients and increase disease awareness. An example is the MASK-air app, which demonstrates how mobile health (mHealth) can advance scientific knowledge and enhance clinical understanding of chronic diseases. The data collected from the app have led to the development of 2 digital biomarkers for daily monitoring of rhinitis and asthma, a better understanding of patient behavior in relation to their diseases, increased knowledge of allergy phenotypes, and quantification of the impact of rhinitis and asthma on quality of life and productivity.^93^ The pharmacist should suggest continuous monitoring of allergic rhinitis through a mobile health app (eg, the MASK-air app). The physician or pharmacist can then review the recorded data, together with the patient, to determine whether any adjustments to the ongoing treatment are necessary.^3^^,^^92^^,^^96^

### The pharmacist in the multidisciplinary network

Multiple studies have emphasized the significant positive impact a pharmacist's good understanding of respiratory conditions can have on their management.^89^^,^^95^^,^^96^ Due to their proximity and accessibility, pharmacists can play a crucial role in helping patients better manage their symptoms. It is paramount to emphasize that pharmacists should not act as substitutes for physicians or other healthcare providers. Instead, they should collaborate closely to ensure the implementation of more timely and personalized management strategies for the specific medical condition. This collaborative approach can significantly enhance clinical outcomes, improve the quality of life for patients, and lead to reductions in direct and indirect healthcare costs.

**Fig. 2:**
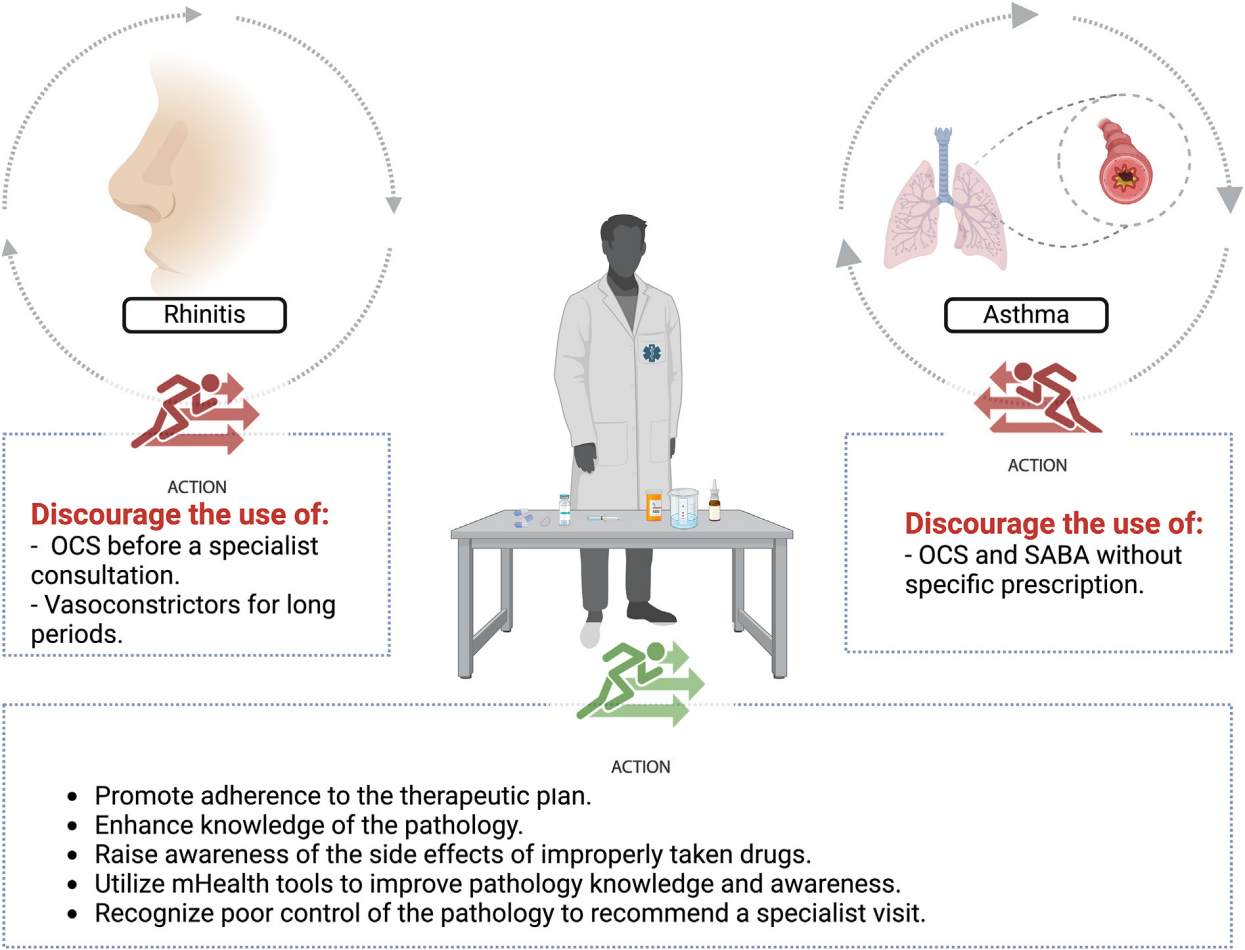
The figure summarizes how the pharmacist can promote and improve the management of asthmatic and rhinitic patients through a direct approach with them. This figure was made with BioRender.com

The pharmacist can therefore improve the patient journey of those suffering from asthma and/or allergic rhinitis in aspects that have been summarized in Fig. 2.

In Italy, structured communication between pharmacists, primary care physicians, and specialists is still evolving. However, emerging models based on shared electronic health records, digital prescribing systems, and integrated care pathways could facilitate more effective interdisciplinary collaboration in the near future.

## Benefits from the increased inclusion of pharmacists in the management of asthmatic and rhinitis patients

The involvement of pharmacists in managing patients with asthma and allergic rhinitis is both possible and desirable, offering several advantages in terms of primary and secondary prevention, as evidenced by a growing body of literature.^72^^,^^105^^,^^106^ The importance of pharmacists in providing direct healthcare, including educational and therapeutic roles within an integrated healthcare system, is also emphasized by the “National Governors Association” and the NHLBI (National Heart, Lung, and Blood Institute) guidelines.^107^^,^^108^ A 2009 literature review, which included 25 studies, demonstrated that pharmacist interventions improve patient outcomes in asthma management, particularly regarding disease severity and quality of life.^109^

Pharmacists are ideally positioned to assist patients in managing asthma due to their clinical expertise and frequent, ongoing contact with affected individuals.^72^^,^^106^ Continuity of care enables easy monitoring of therapy adherence and progression.^105^^,^^110^ Reduced therapeutic compliance, defined as the method and frequency of medication intake and reported in up to 70% of asthma cases, is the primary cause of treatment failure, leading to decreased quality of life and increased healthcare costs.^111^

A notable example comes from Finland, where the Finnish Asthma Programme (1994–2004)^112^ and the Finnish Allergy Programme (2008–2018)^113^ assigned a clear and structured role to community pharmacies. Pharmacists were responsible for checking inhalation techniques, ensuring patients understood the distinction between bronchodilators and inhaled corticosteroids, and encouraging self-monitoring with peak-flow meters. These initiatives, including the Asthma Barometer follow-up study, demonstrated significant improvements in disease control and reduced the societal burden of asthma and allergy. This model highlights the potential value of integrating pharmacists into structured national strategies.

In the United States, the economic impact of non-adherence to therapy exceeds $100 billion, while in Europe it is estimated that poor asthma control increases annual healthcare costs by €1772 per patient.^114^^,^^115^ Similarly, AR presents significant global costs due to its high prevalence, with estimated costs of $3·4 billion in the United States in the early 2000s.^32^^,^^84^

It has been shown that personalized educational interventions by pharmacists are effective, resulting in higher therapy adherence, with sustained compliance (up to 40% more patients maintain adequate compliance after 6 months).^72^^,^^114^ Pharmacists can also oversee the use of over-the-counter (OTC) medications. In this case, beyond ensuring therapeutic compliance, they can guide patients towards the most appropriate therapy, reducing the use of suboptimal drugs and potential side effects (eg, sympathomimetic amines present in topical nasal sprays, frequently used for self-medication in allergic rhinitis).^40^^,^^116^

Therefore, pharmacists can continuously and proactively engage to implement best healthcare practices and achieve optimal clinical outcomes.

## Availability of data and materials and ethics approval

Data not required as a literature review.

## Author contributions

Giovanni Paoletti: conceptualisation, data curation, visualisation writing – original draft. Corrado Giua: methodology, writing – original draft. Alessandro Marti: writing – original draft. Matteo Alberto Baio: writing – original draft. Nicolò Valli: writing – original draft, editing. Erminia Ridolo: writing – review & editing. Maria Teresa Ventura: writing – review & editing. Giovanni Passalacqua: writing – review & editing. Francesca Puggioni: writing – review & editing. Olga Lourenço: writing – review & editing. Jean Bousquet: supervision, writing – review & editing. Giorgio Walter Canonica: supervision, writing – review & editing. Enrico Heffler: writing – review & editing. Carlo Lombardi: supervision, project administration, writing – review & editing.

## Author consent for publication

All authors have confirmed that they have read and approved the manuscript's contents for publication.

## Funding

The publication fee for this work was covered by the Italian Ministry of Heath's 'Ricerca Corrente' funding to the IRCCS Humanitas Research Hospital.

## Declaration of competing interest

G.P. reports personal fees from LoFarma, GSK, Astrazeneca outside the submitted work, E. R. reports personal fees from Menarini international outside the submitted work, M.T.V. reports personal fees from Member of the board of Italian Society of Allergology, Asthma and Clinical Immunology President of the Medical Women's Italian Association- Bari outside the submitted work, F.P. reports personal fees from Sanofi, gsk, menarini, regeneron, stallergen greer, alk abello, chiesi, astrazenenca outside the submitted work, J.B.r reports personal fees from Cipla, Menarini, Mylan, Novartis, Purina, Sanofi-Aventis, Teva, Noucor, others from KYomed-Innov, others from Mask-air-SAS, outside the submitted work., G.W.C. reports personal fees from Menarini, AstraZeneca, CellTrion, Chiesi, Faes Farma, Firma, Genentech, Guidotti-Malesci, GSK, HAL Allergy, Innovacaremd, Novartis, OM-Pharma, Red Maple, Sanofi-Aventis, Sanofi-Genzyme, Stallergenes-Greer and Uriach Pharma. outside the submitted work, E.H. reports personal fees from Chiesi, Astrazeneca, Apogee therapeutics, Celltrion Healthcare, GSK, Almirall, Sanofi.

Regeneron Firma, Novartis, Lofarma outside the submitted work C.L. reports personal fees from Menarini, AstraZeneca, Chiesi, Firma, GSK, Sanofi-Genzyme. outside the submitted work. A.M., N.V., G.Pas., M.A.B., C.G., O.L. don't report personal fees to declare.

## References

[1] Bousquet J., Khaltaev N., Cruz A.A. (2008). Allergic Rhinitis and its impact on Asthma (ARIA) 2008. Allergy.

[2] Passalacqua G. (2000). United airways disease: therapeutic aspects. Thorax.

[3] Bousquet J., Schünemann H.J., Togias A. (2020). Next-generation allergic Rhinitis and its impact on Asthma (ARIA) guidelines for allergic rhinitis based on grading of recommendations assessment, development and evaluation (GRADE) and real-world evidence. J Allergy Clin Immunol.

[4] Nappi E., Paoletti G., Malvezzi L. (2022). Comorbid allergic rhinitis and asthma: important clinical considerations. Expet Rev Clin Immunol.

[5] Bousquet J., Toumi M., Sousa-Pinto B. (2022). The allergic rhinitis and its impact on asthma (ARIA) approach of value-added medicines: as-needed treatment in allergic rhinitis. J Allergy Clin Immunol Pract.

[6] Reddel H.K., Taylor D.R., Bateman E.D. (2009). An official American thoracic society/European respiratory society statement: asthma control and exacerbations. Am J Respir Crit Care Med.

[7] Bousquet P.-J., Leynaert B., Neukirch F. (2008). Geographical distribution of atopic rhinitis in the european community respiratory health survey I. Allergy.

[8] Bousquet J., Anto J.M., Bachert C. (2020). Allergic rhinitis. Nat Rev Dis Primers.

[9] Savouré M., Bousquet J., Jaakkola J.J.K., Jaakkola M.S., Jacquemin B., Nadif R. (2022). Worldwide prevalence of rhinitis in adults: a review of definitions and temporal evolution. Clin Transl Allergy.

[10] Licari A., Magri P., De Silvestri A. (2023). Epidemiology of allergic rhinitis in children: a systematic review and meta-analysis. J Allergy Clin Immunol Pract.

[11] Olivieri M., Verlato G., Corsico A. (2002). Prevalence and features of allergic rhinitis in Italy. Allergy.

[12] Mantovani L.G., Bettoncelli G., Cricelli C. (2007). Allergic rhinitis in the Italian population evaluated through the national database of general practitioners. Allergy.

[13] de Marco R., Cappa V., Accordini S. (2012). Trends in the prevalence of asthma and allergic rhinitis in Italy between 1991 and 2010. Eur Respir J.

[14] CGHSLRBM Pawankar R. (2013). WAO White Book on Allergy: 2013 Update.

[15] Sun W., Ding C., Jiang Z., Zheng X., Jiang J., Xu H. (2024). The impact of ambient air pollution on allergic rhinitis symptoms: a prospective Follow-Up study. Toxics.

[16] Zhong J., Li W., Yang S., Shen Y., Li X. (2024). Causal association between air pollution and allergic rhinitis, asthma: a Mendelian randomization study. Front Public Health.

[17] Eguiluz-Gracia I., Mathioudakis A.G., Bartel S. (2020). The need for clean air: the way air pollution and climate change affect allergic rhinitis and asthma. Allergy.

[18] Scelo G., Torres-Duque C.A., Maspero J. (2024). Analysis of comorbidities and multimorbidity in adult patients in the international severe asthma registry. Ann Allergy Asthma Immunol.

[19] Thompson A.K., Juniper E., Meltzer E.O. (2000). Quality of life in patients with allergic rhinitis. Ann Allergy Asthma Immunol.

[20] Vieira R.J., Pham-Thi N., Anto J.M. (2022). Academic productivity of young people with allergic rhinitis: a MASK-Air study. J Allergy Clin Immunol Pract.

[21] Vandenplas O., Vinnikov D., Blanc P.D. (2018). Impact of rhinitis on work productivity: a systematic review. J Allergy Clin Immunol Pract.

[22] Cardell L.-O., Olsson P., Andersson M. (2016). TOTALL: high cost of allergic Rhinitis—a national Swedish population-based questionnaire study. NPJ Prim Care Respir Med.

[23] Rodrigues J., Franco-Pego F., Sousa-Pinto B., Bousquet J., Raemdonck K., Vaz R. (2021). Anxiety and depression risk in patients with allergic rhinitis: a systematic review and meta-analysis. Rhinology journal.

[24] Giuliano A.F.M., Buquicchio R., Patella V. (2022). Rediscovering allergic rhinitis: the use of a novel mHealth solution to describe and monitor health-related quality of life in elderly patients. Int Arch Allergy Immunol.

[25] Ventura M.T., Giuliano A.F.M., Buquicchio R. (2022). Implementation of the MASK-Air® app for rhinitis and asthma in older adults: mask@puglia pilot study. Int Arch Allergy Immunol.

[26] Lombardi C., Asero R., Bagnasco D. (2021). ARIA-ITALY multidisciplinary consensus on nasal polyposis and biological treatments. World Allergy Organization Journal.

[27] Canonica G.W., Colombo G.L., Bruno G.M. (2019). Shadow cost of oral corticosteroids-related adverse events: a pharmacoeconomic evaluation applied to real-life data from the severe asthma network in Italy (SANI) registry. World Allergy Organization Journal.

[28] Patella V., Florio G., Magliacane D. (2019). Public prevention plans to manage climate change and respiratory allergic diseases. Innovative models used in Campania region (Italy): the twinning aria implementation and the allergy safe tree decalogue. Transl Med UniSa.

[29] Rodriguez A., Brickley E., Rodrigues L., Normansell R.A., Barreto M., Cooper P.J. (2019). Urbanisation and asthma in low-income and middle-income countries: a systematic review of the urban–rural differences in asthma prevalence. Thorax.

[30] Bousquet J.J., Schünemann H.J., Togias A. (2019). Next-generation ARIA care pathways for rhinitis and asthma: a model for multimorbid chronic diseases. Clin Transl Allergy.

[31] Bosnic-Anticevich S., Costa E., Menditto E. (2019). <scp>ARIA</scp> pharmacy 2018 “Allergic rhinitis care pathways for community pharmacy”. Allergy.

[32] Lourenço O., Cvetkovski B., Kritikos V. (2022). Management of allergic rhinitis symptoms in the pharmacy pocket guide 2022. Clin Transl Allergy.

[33] Members of the Workshops (2004). ARIA in the pharmacy: management of allergic rhinitis symptoms in the pharmacy. Allergic rhinitis and its impact on asthma. Allergy.

[34] Lall D., Engel N., Devadasan N., Horstman K., Criel B. (2018). Models of care for chronic conditions in low/middle-income countries: a ‘best fit’ framework synthesis. BMJ Glob Health.

[35] Giulio De Belvis A., Meregaglia M., Morsella A. (2022). Health system review Italy. Health Systems in Transition: Italy.

[36] Heffler E., Madeira L.N.G., Ferrando M. (2018). Inhaled corticosteroids safety and adverse effects in patients with asthma. J Allergy Clin Immunol Pract.

[37] Heffler E., Bagnasco D., Canonica G.W. (2019). Strategies to reduce corticosteroid-related adverse events in asthma. Curr Opin Allergy Clin Immunol.

[38] Scheire S., Germonpré S., Mehuys E. (2024). Rhinitis control and medication use in a real-world sample of patients with persistent rhinitis or rhinosinusitis: a community pharmacy study. J Allergy Clin Immunol Pract.

[39] Mokhatrish M., Almatrafi S., Aldrees T. (2024). Pharmacists' attitudes towards long-term use of nasal decongestants: a cross-sectional study. J Multidiscip Healthc.

[40] Russo E., Giombi F., Paoletti G. (2023). Use, abuse, and misuse of nasal medications: real-life survey on community pharmacist's perceptions. J Personalized Med.

[41] Federchimica Assosalute (2023).

[42] Baena-Cagnani C.E., Canonica G.W., Zaky Helal M. (2015). The international survey on the management of allergic rhinitis by physicians and patients (ISMAR). World Allergy Organization Journal.

[43] Price D., Scadding G., Ryan D. (2015). The hidden burden of adult allergic rhinitis: UK healthcare resource utilisation survey. Clin Transl Allergy.

[44] Manjit Singh P.K., Krishnan E.K., Mat Lazim N., Yaacob N.M., Abdullah B. (2022). Medication adherence to intranasal corticosteroids in allergic rhinitis patients with comorbid medical conditions. Pharmaceutics.

[45] Canonica G.W., Domingo C., Lavoie K.L. (2024). Asthma patients' and physicians' perspectives on the burden and management of asthma: post-hoc analysis of APPaRENT 1 and 2 to assess predictors of treatment adherence. Respir Med.

[46] Gregoriano C., Dieterle T., Breitenstein A.-L. (2018). Use and inhalation technique of inhaled medication in patients with asthma and COPD: data from a randomized controlled trial. Respir Res.

[47] Pharmaceutical Group of the European Union (PGEU) (2019).

[48] Federfarma (2024).

[49] Tharumia Jagadeesan C., Wirtz V.J. (2021). Geographical accessibility of medicines: a systematic literature review of pharmacy mapping. J Pharm Policy Pract.

[50] (2012). DECRETO-LEGGE 24 Gennaio 2012.

[51] Berenbrok L.A., Tang S., Gabriel N. (2022). Access to community pharmacies: a nationwide geographic information systems cross-sectional analysis. J Am Pharmaceut Assoc.

[52] Valliant S.N., Burbage S.C., Pathak S., Urick B.Y. (2022). Pharmacists as accessible health care providers: quantifying the opportunity. J Manag Care Spec Pharm.

[53] Giua C., Paoletti G., Minerba L. (2021). Community pharmacist's professional adaptation amid Covid-19 emergency: a national survey on Italian pharmacists. Int J Clin Pharm.

[54] Agenzia Nazionale per i Servizi Sanitari Regionali (2024).

[55] Agenzia Nazionale per i Servizi Sanitari Regionali (2023).

[56] Pharmaceutical Group of the European Union (PGEU) (2016).

[57] (2018). The changing role of the pharmacist in the 21st century. Pharm J.

[58] Bragazzi N., Mansour M., Bonsignore A., Ciliberti R. (2020). The role of hospital and community pharmacists in the management of COVID-19: towards an expanded definition of the roles, responsibilities, and duties of the pharmacist. Pharmacy.

[59] Giua C., Minghetti P., Gandolini G. (2020). Community pharmacist's role in detecting low back pain, and patient attitudes—A cross-sectional observational study in Italian community pharmacies. Int J Environ Res Publ Health.

[60] El-Den S., Lee Y.L.E., Gide D.N., O'Reilly C.L. (2022). Stakeholders' acceptability of pharmacist-led screening in community pharmacies: a systematic review. Am J Prev Med.

[61] Paudyal V., Fialová D., Henman M.C. (2021). Pharmacists' involvement in COVID-19 vaccination across Europe: a situational analysis of current practice and policy. Int J Clin Pharm.

[62] Baratta F., Enri L.R., Brusa P. (2023). Community pharmacists as vaccinators in the Italian SARS-CoV-2 immunization campaign: implications beyond the pandemic. Health Policy.

[63] Euforea. Euforea exchange. https://euforea-exchange.com/taxonomy/term/29?page=4.

[64] Pearson G.J. (2007). Evolution in the practice of pharmacy--not a revolution. Can Med Assoc J.

[65] Dreischulte T., van den Bemt B., Steurbaut S. (2022). European society of clinical pharmacy definition of the term clinical pharmacy and its relationship to pharmaceutical care: a position paper. Int J Clin Pharm.

[66] Moltó-Puigmartí C., Vonk R., van Ommeren G., Hegger I. (2018). A logic model for pharmaceutical care. J Health Serv Res Policy.

[67] Brunetti P., Baldessin L., Pagliacci S. (2022). Prediabetes, undiagnosed diabetes and diabetes risk in Italy in 2017–2018: results from the first National screening campaign in community pharmacies. J Public Health.

[68] Spadea T., Onorati R., Baratta F. (2021). Monitoring adherence to pharmacological therapy and follow-up examinations among patients with type 2 diabetes in community pharmacies. Results from an experience in Italy. PLoS One.

[69] Omboni S., Caserini M. (2018). Effectiveness of pharmacist's intervention in the management of cardiovascular diseases. Open Heart.

[70] Omboni S., Tenti M., Coronetti C. (2019). Physician–pharmacist collaborative practice and telehealth may transform hypertension management. J Hum Hypertens.

[71] Giua C., Minerba L., Piras A. (2022). Save Your Heart - studio osservazionale trasversale, multicentrico, italiano, sulla presenza di fattori di rischio cardiovascolare in partecipanti affetti da ipertensione. Giornale Italiano di Health Technology Assessment Delivery.

[72] Paoletti G., Keber E., Heffler E. (2020). Effect of an educational intervention delivered by pharmacists on adherence to treatment, disease control and lung function in patients with asthma. Respir Med.

[73] Pasina L., Urru S.A.M., Minghetti P., Giua C. (2015). Role of community pharmacies for the detection of potentially inappropriate Xanthine oxidase inhibitor prescriptions. Drugs Real World Outcomes.

[74] Urru S.A.M., Pasina L., Minghetti P., Giua C. (2015). Role of community pharmacists in the detection of potentially inappropriate benzodiazepines prescriptions for insomnia. Int J Clin Pharm.

[75] Pasina L., Urru S.A.M., Mandelli S., Giua C., Minghetti P. (2016). Evidence-based and unlicensed indications for proton pump inhibitors and patients' preferences for discontinuation: a pilot study in a sample of Italian community pharmacies. J Clin Pharm Therapeut.

[76] Kwan∗ J.L., Lo∗ L., Sampson M., Shojania K.G. (2013). Medication reconciliation during transitions of care as a patient safety strategy. Ann Intern Med.

[77] Brusa P., Allais G., Scarinzi C. (2019). Self-medication for migraine: a nationwide cross-sectional study in Italy. PLoS One.

[78] Keber E., Rocco P., Musazzi U.M. (2021). The management of upper gastrointestinal symptoms: a study on community pharmacies in Italy. Pharmacia.

[79] Giua C., Romano F., Keber E. (2024). A prospective real-world study of Bacillus clausii evaluating use, treatment habits and patient satisfaction in Italian community pharmacies: the PEGASO study. Drugs Real World Outcomes.

[80] Giua C., Minerba L., Piras A. (2021). The effect of sucralfate-containing ointment on quality of life in people with symptoms associated with haemorrhoidal disease and its complications: the results of the EMOCARE survey. Acta Biomed.

[81] Chew C.C., Chang C.T., Lim X.J. (2022). The management of allergic rhinitis by pharmacists in public services: a proposed PhaRmacISt-led education model (AR-PRISE). J Pharm Policy Pract.

[82] Canonica G.W., Triggiani M., Senna G.E. (2015). 360 degree perspective on allergic rhinitis management in Italy: a survey of GPs, pharmacists and patients. Clin Mol Allergy.

[83] Kuehl B.L., Abdulnour S., O’dell M., Kyle T.K. (2015). Understanding the role of the healthcare professional in patient self-management of allergic rhinitis. SAGE Open Med.

[84] May J.R., Dolen W.K. (2017). Management of allergic rhinitis: a review for the community pharmacist. Clin Ther.

[85] Tan R., Cvetkovski B., Kritikos V. (2018). The burden of rhinitis and the impact of medication management within the community pharmacy setting. J Allergy Clin Immunol Pract.

[86] Klimek L., Bachert C., Pfaar O. (2019). ARIA guideline 2019: treatment of allergic rhinitis in the German health system. Allergol Select.

[87] José J., Cvetkovski B., Kritikos V., Tan R., Bosnic-Anticevich S., Lourenço O. (2020). Interventions delivered in the community pharmacy to manage allergic Rhinitis- A systematic review of the literature. Pharmacy.

[88] Wojas O., Krzych-Fałta E., Furmańczyk K., Sybilski A., Lisiecka-Biełanowicz M., Samoliński B. (2019). The use of nasal over-the-counter agents in the evaluated Polish population. The underrated role of the pharmacist in patient education on medical treatment in patients with allergic rhinitis. Postepy Dermatol Alergol.

[89] Lourenço O., Bosnic-Anticevich S., Costa E. (2020). Managing allergic rhinitis in the pharmacy: an ARIA guide for implementation in practice. Pharmacy.

[90] Nappi E., Keber E., Paoletti G. (2023). Oral corticosteroid abuse and self-prescription in Italy: a perspective from community pharmacists and sales reports before and during the COVID-19 era. J Personalized Med.

[91] Chawhan A., Thakrar D., Pinto L. (2023). Inhaler devices and their challenges - helping patients use inhalers. Lung India.

[92] Szylling A., Raciborski F., Wojas O. (2023). Why the role of mHealth in allergy diagnosis and treatment adherence cannot be overlooked. Clin Transl Allergy.

[93] Sousa-Pinto B., Fonseca J., Bousquet J. (2024). Contribution of MASK-air® as an mHealth tool for digitally enabled person-centered care in rhinitis and asthma. Journal of Investigational Allergy and Clinical Immunology.

[94] Bousquet J., Hellings P.W., Agache I. (2016). Aria 2016: care pathways implementing emerging technologies for predictive medicine in rhinitis and asthma across the life cycle. Clin Transl Allergy.

[95] Chisholm-Burns M.A., Kim Lee J., Spivey C.A. (2010). US pharmacists' effect as team members on patient care: systematic review and meta-analyses. Med Care.

[96] Bosnic-Anticevich S., Costa E., Menditto E. (2019). ARIA pharmacy 2018 “allergic rhinitis care pathways for community pharmacy”: AIRWAYS ICPs initiative (European innovation partnership on active and healthy ageing, DG CONNECT and DG santé) POLLAR (impact of air POLLution on asthma and rhinitis) GARD demonstration project. Allergy: European Journal of Allergy and Clinical Immunology.

[97] Wheatley L.M., Togias A. (2015). Clinical practice. Allergic rhinitis. N Engl J Med.

[98] Bousquet J., Melén E., Haahtela T. (2023). Rhinitis associated with asthma is distinct from rhinitis alone: the ARIA-MeDALL hypothesis. Allergy: European Journal of Allergy and Clinical Immunology.

[99] Klimek L., Bergmann K.C., Biedermann T. (2017). Visual analogue scales (VAS) - measuring instruments for the documentation of symptoms and therapy monitoring in case of allergic rhinitis in everyday health care. Allergo J.

[100] Wise S.K., Damask C., Roland L.T. (2023). International consensus statement on allergy and rhinology: allergic rhinitis – 2023. Int Forum Allergy Rhinol.

[101] Chew C.C., Lim X.J., Letchumanan P., Narayanan M.S., Rajan P., Chong C.P. (2023). Development and validation of a pharmacist-led education model in allergic rhinitis management: a multi-phase study. J Pharm Policy Pract.

[102] Hamelmann E., Hammerby E., Scharling K.S., Pedersen M., Okkels A., Schmitt J. (2024). Quantifying the benefits of early sublingual allergen immunotherapy tablet initiation in children. Allergy.

[103] Sahiner U.M., Giovannini M., Escribese M.M. (2023). Mechanisms of allergen immunotherapy and potential biomarkers for clinical evaluation. J Personalized Med.

[104] Paoletti G., Di Bona D., Chu D.K. (2021). Allergen immunotherapy: the growing role of observational and randomized trial “Real-World Evidence”. Allergy.

[105] Bridgeman M.B., Wilken L.A. (2021). Essential role of pharmacists in asthma care and management. J Pharm Pract.

[106] Putman B., Coucke L., Vanoverschelde A., Mehuys E., Lahousse L. (2022). Community pharmacist counseling improves adherence and asthma control: a nationwide study. BMC Health Serv Res.

[107] Isasi F., Krofah E. (2015).

[108] National Heart L and BInstituteN asthma education and prevention program (2007). Expert panel report 3 (EPR-3): guidelines for the diagnosis and management of asthma–summary report 2007. J Allergy Clin Immunol.

[109] Benavides S., Rodriguez J.C., Maniscalco-Feichtl M. (2009). Pharmacist involvement in improving asthma outcomes in various healthcare settings: 1997 to present. Ann Pharmacother.

[110] Makhinova T., Barner J.C., Brown C.M., Richards K.M., Rascati K.L., Nag A. (2022). Improving asthma management: patient–pharmacist partnership program in enhancing therapy adherence. Pharmacy.

[111] Baiardini I., Paoletti G., Malipiero G. (2020). Validation of the Italian version of the test of adherence to inhalers. J Investig Allergol Clin Immunol.

[112] Haahtela T. (2006). A 10 year asthma programme in Finland: major change for the better. Thorax.

[113] Haahtela T., Valovirta E., Saarinen K. (2021). The Finnish allergy program 2008-2018: society-Wide proactive program for change of management to mitigate allergy burden. J Allergy Clin Immunol.

[114] Manfrin A., Tinelli M., Thomas T., Krska J. (2017). A cluster randomised control trial to evaluate the effectiveness and cost-effectiveness of the Italian medicines use review (I-MUR) for asthma patients. BMC Health Serv Res.

[115] Dierick B.J.H., van der Molen T., Flokstra-de Blok B.M.J. (2020). Burden and socioeconomics of asthma, allergic rhinitis, atopic dermatitis and food allergy. Expert Rev Pharmacoecon Outcomes Res.

[116] Brożek J.L., Bousquet J., Agache I. (2017). Allergic rhinitis and its impact on asthma (ARIA) guidelines—2016 revision. J Allergy Clin Immunol.

